# Redefining Cystic Fibrosis-Related Diabetes Management With Tirzepatide: A Case Report

**DOI:** 10.7759/cureus.97448

**Published:** 2025-11-21

**Authors:** Yashwanth Suresh Babu, Vijay Jayagopal

**Affiliations:** 1 Internal Medicine, York Hospital, York and Scarborough Teaching Hospitals NHS Foundation Trust, York, GBR; 2 Diabetes and Endocrinology, York Hospital, York and Scarborough Teaching Hospitals NHS Foundation Trust, York, GBR

**Keywords:** ‘cystic fibrosis’, cystic fibrosis-related diabetes, glycaemic control, metabolic syndrome, tirzepatide

## Abstract

Cystic fibrosis-related diabetes (CFRD) is the most common comorbidity in adults with cystic fibrosis (CF). Traditional management relied on insulin therapy to counter the rise in glucose related to a carbohydrate-rich diet, which was previously recommended. Disease-modifying therapies for CF have, however, changed the metabolic milieu, and we are seeing rising rates of people with CF experiencing weight gain and developing metabolic syndrome. This calls for a paradigm shift in managing CFRD.

We describe a 51-year-old woman with long-standing CFRD who, despite basal-bolus insulin, had suboptimal glycaemic control and emerging cardiovascular risk factors. After initiation of tirzepatide, she experienced improved glucose control, HbA1c, weight and blood pressure over three months without major adverse effects.

This case illustrates the potential of tirzepatide to address both glycaemic and cardiometabolic risk in CFRD and highlights the need for formal evaluation of GLP-1/GIP receptor agonists in this population.

## Introduction

Cystic fibrosis (CF) is an autosomal recessive condition caused by mutations in the CF transmembrane conductance regulator (CFTR) gene, which encodes a chloride/bicarbonate channel expressed in airway, intestinal and exocrine tissues [1,2]. With the advent of highly effective modulator therapies (HEMTs), life expectancy for people with CF has improved dramatically [3,4].

Among the major non-pulmonary complications is cystic fibrosis-related diabetes (CFRD), which affects up to 40-50 % of adults with CF [5]. Historically, nutritional guidance for CF emphasised a high-fat, high-calorie “legacy” diet to prevent malnutrition and preserve lung function [6]. However, as life expectancy extends and modulator therapies lead to improved nutrition and body composition, the prevalence of overweight and obesity in CF is rising, for example, in one US dataset, overweight jumped >300 % and obesity >400 % between 2000 and 2019 [7]. This reflects an emerging phenotype of “CFRD with metabolic syndrome” in which traditional cardiovascular risk factors (hypertension, dyslipidaemia, central adiposity) now appear in a population previously at low risk.

Mechanistically, multiple factors contribute to the development of metabolic syndrome in CF, including chronic systemic inflammation, CFTR-related alterations in adipose and skeletal muscle metabolism, reduced physical activity, and the continued use of high-calorie “legacy” diets. CFTR modulators may further influence energy balance and adiposity, contributing to excess weight gain in some patients [8].

CFRD differs pathophysiologically from both type 1 and type 2 diabetes. Unlike type 1 diabetes, it is not autoimmune, and unlike type 2 diabetes, β-cell dysfunction rather than primary insulin resistance predominates. Pancreatic fibrosis, islet inflammation, and loss of β-cell mass cause impaired insulin secretion, while concurrent insulin resistance - exacerbated by obesity, infection, or corticosteroid use - is increasingly recognised in the modern CF population [9,10]. Consequently, insulin therapy alone may not adequately address the dual burden of insulin deficiency and resistance. Previous pharmacological attempts beyond insulin, such as GLP-1 receptor agonists, have shown potential to improve glucose tolerance and postprandial insulin secretion in CFRD but have been limited by gastrointestinal side effects and modest efficacy [11].

A dual GIP/GLP-1 receptor agonist, such as tirzepatide, may be mechanistically suited to this phenotype: GLP-1 receptor activation enhances glucose-dependent insulin secretion, suppresses glucagon, and promotes weight loss, while GIP agonism may further improve insulin secretion and modulate adipose metabolism [12,13]. Together, these effects could target both impaired β-cell function and insulin resistance in CFRD with metabolic syndrome. We report a case illustrating the potential role of tirzepatide in managing CFRD with coexisting metabolic syndrome.

## Case presentation

A 51-year-old woman with CF (homozygous F508del) and CFRD, diagnosed 25 years earlier, was reviewed in March 2025. She had been established on Elexacaftor/Tezacaftor/Ivacaftor for five years with stable pulmonary function. Her major ongoing issue remained poor glycaemic control.

**Figure 1:**
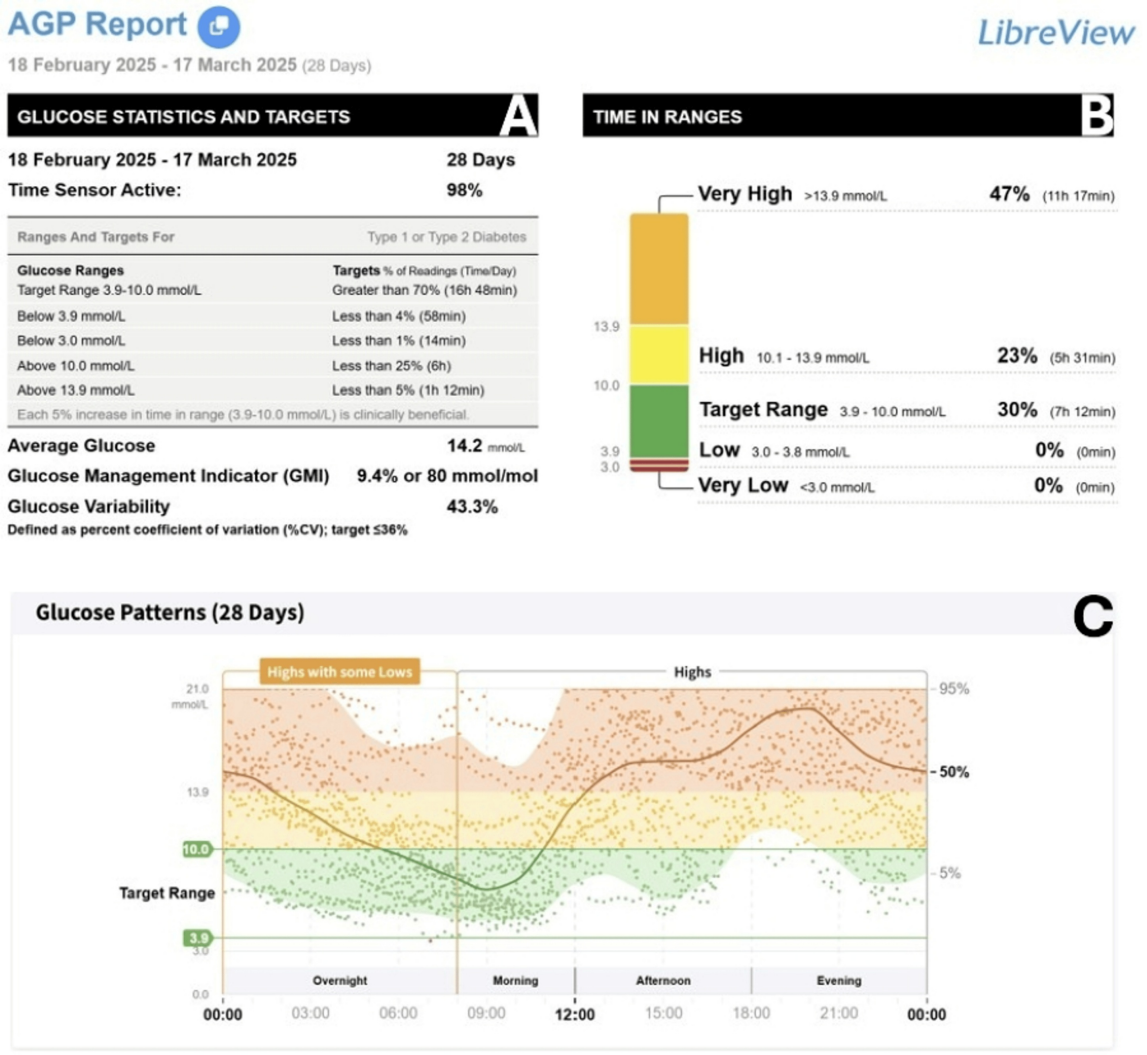
Continuous glucose monitoring data in the 28 days prior to starting Tirzepatide. (A) Glucose statistics and targets. (B) Time in range. (C) Glucose pattern over 24 hours. Orange correlates with glucose in the very high range, yellow correlates with glucose in the high range, green correlates with glucose within the target range, red correlates with glucose in the low range, and maroon correlates with glucose in the very low range.

At baseline, she was managed with basal-bolus insulin therapy (insulin glargine 8 units each morning with prandial insulin aspart). Despite this, her mean glucose over the preceding four weeks, as recorded by real-time continuous glucose monitoring (CGM), was 14.2 mmol/L, with a time in range (TIR) of 30% (Figure 1). As portrayed in Figure 1C, apart from a few hours in the morning, her glucose was in the high or very high range for the majority of the day. Her blood pressure was 145/87 mmHg, weight 65.8 kg, and BMI 25.7 kg/m² (Table 1). Laboratory evaluation revealed an intermediate C-peptide level. Her most recent HbA1c and total cholesterol, measured in December 2024, were 70 mmol/mol and 3.1 mmol/L, respectively. After discussion of potential risks and benefits, she consented to an off-label trial of tirzepatide 2.5 mg once weekly.

**Table 1:** Weight, BMI, and blood pressure over time.

Parameter	March 2025	April 2025	May 2025
Weight (kg)	65.8	64.4	61.8
BMI (kg/m²)	25.7	25.2	24.1
BP Systolic (mmHg)	145	147	127
BP Diastolic (mmHg)	87	91	88

At follow-up in April 2025, tirzepatide was noted to be well-tolerated and was uptitrated to 5 mg weekly. Dapagliflozin 10 mg once daily was also initiated. Her HbA1c at this time was 65 mmol/mol (Table 2).

**Table 2:** HbA1c over time. Note that tirzepatide 2.5 mg/weekly was initiated in March 2025, uptitrated to 5 mg/weekly in April 2025 and then uptitrated to 7.5 mg/weekly in May 2025.

Date	HbA1c (mmol/mol)
December 2024	65
April 2025	65
May 2025	62
June 2025	64

By May 2025, she had lost 4 kg, with BMI decreasing from 25.7 to 24.1 kg/m², and systolic blood pressure improving from 145 to 127 mmHg (Table 1). Her HbA1c improved to 62 mmol/mol, representing her best glycaemic control in several years (Table 2). Tirzepatide was subsequently increased to 7.5 mg weekly.

In June 2025, her total cholesterol remained within the normal range at 3.7 mmol/L. By July, she reported feeling well, with transient nausea resolving and constipation managed conservatively. No significant adverse effects were observed.

**Figure 2:**
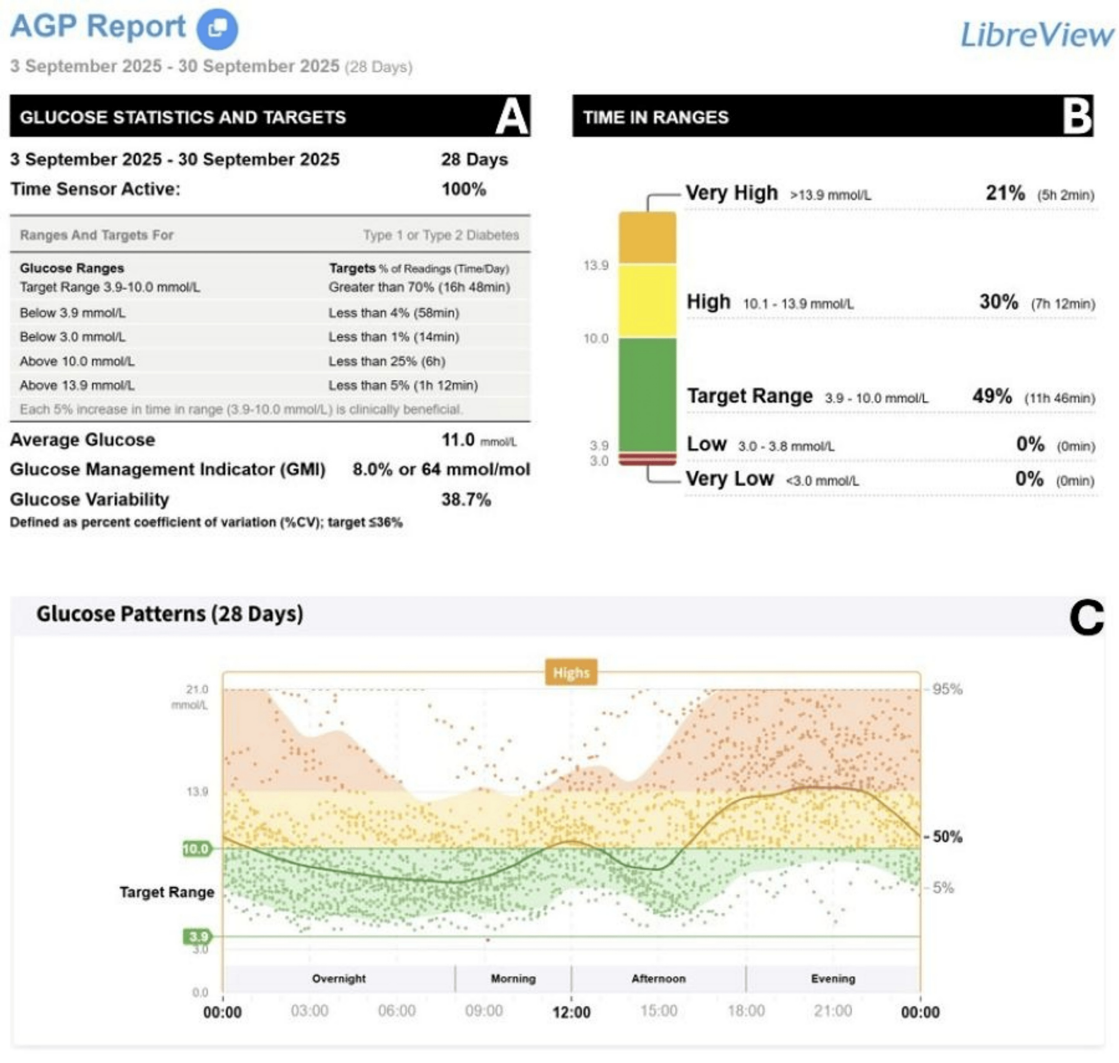
Continuous glucose monitoring data in 28 days prior to the six-month mark of starting tirzepatide. (A) Glucose statistics and targets. (B) Time in range. (C) Glucose pattern over 24 hours. Orange correlates with glucose in the very high range, yellow correlates with glucose in the high range, green correlates with glucose within the target range, red correlates with glucose in the low range, and maroon correlates with glucose in the very low range.

At her six-month review in September 2025, her mean glucose had improved to 11 mmol/L over four weeks with a TIR of 49% (Figure 2). As seen in Figure 2C, her glucose pattern improved, with it being in the target range throughout the night up till the afternoon. Her insulin requirements were markedly reduced; she remained on dapagliflozin with only 6 units of basal insulin daily and no longer required prandial insulin correction.

Overall, initiation of tirzepatide with the addition of dapagliflozin led to objective improvements in glycaemic control, body weight, and blood pressure, alongside a substantial reduction in insulin requirements.

## Discussion

Obesity is emerging in CF populations as life expectancy rises and CF is better managed with less catabolic demand. Although the Cystic Fibrosis Foundation still recommends BMI ≥22 kg/m² in adult females and ≥23 kg/m² in adult males [14], some patients now exceed these targets, introducing cardiovascular risks historically uncommon in CF.

Tirzepatide (LY3298176) is a first-in-class dual GIP and GLP-1 receptor agonist approved for type 2 diabetes and obesity. As mentioned earlier, it combines the incretin effects of both hormones, and emerging evidence also suggests anti-inflammatory and endothelial benefits, which could be particularly relevant in CF, a condition characterised by systemic inflammation and heightened cardiovascular risk [15].

Her intermediate C-peptide level indicates residual β-cell function, consistent with partial pancreatic insufficiency and supporting the mixed pathophysiology of CFRD, in which both insulin deficiency and insulin resistance coexist [16]. This finding differentiates CFRD from type 1 diabetes, where C-peptide is typically undetectable, and from type 2 diabetes, where levels are preserved or elevated. It also underscores the mechanistic rationale for using dual GIP/GLP-1 receptor agonists, which, as mentioned earlier, enhance endogenous insulin secretion while improving insulin sensitivity.

In the SURPASS-2 trial (NEJM, 2021), tirzepatide reduced HbA1c by 2.01-2.30% and body weight by 7.6-11.2 kg after 40 weeks, outperforming semaglutide 1 mg (-1.86% and -5.7 kg, respectively)[17]. In our patient, tirzepatide use was associated with a 4 kg weight loss over 3 months (BMI 25.7 → 24.1 kg/m²) and a modest HbA1c improvement from 65 to 62 mmol/mol at three months, before a slight rise to 64 mmol/mol at three months. Although the glycaemic improvement was smaller than that reported in type 2 diabetes trials, the direction and magnitude of weight change align with early responses observed in SURPASS-2, suggesting a potential translational benefit in CFRD.

Previous pharmacologic attempts beyond insulin, such as selective GLP-1 receptor agonists, have shown modest improvements in glucose tolerance and post-prandial insulin secretion in CFRD [18,19]. However, gastrointestinal side effects and variable efficacy have limited their clinical adoption. The dual incretin mechanism of tirzepatide may therefore offer a mechanistically broader approach, addressing both β-cell dysfunction and insulin resistance. Although tirzepatide was well tolerated in our patient, gastrointestinal symptoms and rare adverse effects such as pancreatitis warrant vigilance in CF, particularly given existing pancreatic pathology.

Given the evolving metabolic profile of CF patients, consideration of newer T2DM therapies such as SGLT2 inhibitors (e.g., dapagliflozin), as used in our study, may also be warranted to optimise metabolic and cardiovascular outcomes.

Limitations of this report include its single-patient design, off-label use, short follow-up, the concurrent use of dapagliflozin as a potential confounding factor, and lack of long-term cardiovascular outcome data. Nevertheless, this case highlights the potential for a novel therapeutic paradigm in CFRD management, addressing not only hyperglycaemia but also obesity and cardiovascular risk. As the CF population continues to evolve metabolically, larger studies are needed to establish the safety, efficacy, and long-term impact of tirzepatide in this unique patient group. There is a need for a paradigm shift in how we consider CFRD, moving from therapy akin to type 1 diabetes to likening the condition more to managing someone with type 2 diabetes.

## Conclusions

As obesity and metabolic syndrome become more prevalent in people with CF, traditional insulin-centric approaches to CFRD may be insufficient. Our case illustrates the potential of tirzepatide to improve glycaemia and cardiometabolic parameters, although the concurrent use of dapagliflozin may have contributed to these effects. Controlled studies are warranted to establish safety and efficacy in this population.
